# Response assessment in metastatic melanoma treated with ipilimumab and bevacizumab: CT tumor size and density as markers for response and outcome

**DOI:** 10.1186/s40425-014-0040-2

**Published:** 2014-11-18

**Authors:** Mizuki Nishino, Anita Giobbie-Hurder, Nikhil H Ramaiya, F Stephen Hodi

**Affiliations:** Department of Radiology, Brigham and Women’s Hospital and Dana-Farber Cancer Institute, 450 Brookline Avenue, Boston, MA 02215 USA; Department of Biostatistics and Computational Biology, Dana-Farber Cancer Institute, 450 Brookline Avenue, Boston, MA 02215 USA; Department of Medical Oncology and Department of Medicine, Dana-Farber Cancer Institute and Brigham and Women’s Hospital, 450 Brookline Avenue, Boston, MA 02215 USA

**Keywords:** Melanoma, Immunotherapy, Anti-angiogenic therapy, Tumor response, Density, RECIST

## Abstract

**Background:**

Investigate the tumor diameter and density changes in advanced melanoma patients treated with ipilimumab plus bevacizumab, compare response rates based on different response criteria, and study association between these measures and survival.

**Methods:**

Twenty-one advanced melanoma patients with 59 measurable lesions treated in a phase 1 trial of ipilimumab plus bevacizumab were retrospectively studied. Tumor diameter and density were measured on baseline and first follow-up CT. Responses were assigned using RECIST, MASS and Choi criteria. Diameter and density measures and responses by these criteria were studied for the association with survival.

**Results:**

Twenty-three (39%) lesions and 7 (33%) patients met the Choi density criteria for response (≥15% density decrease) at the first follow-up. The response rates were 14% (3/21, 95% CI: 3-36%) by RECIST and MASS, and 52% (11/21, 95% CI: 30-74%) by Choi criteria, when both size and density criteria were used. Larger baseline tumor diameter was significantly associated with shorter progression-free survival (PFS) and overall survival (OS) (log-rank p = 0.001 and 0.003; respectively). Diameter or density changes, or responses by RECIST, MASS or Choi criteria at the first follow-up, were not associated with PFS or OS.

**Conclusion:**

Tumor density decrease meeting Choi criteria was noted in one-third of advanced melanoma patients at the first follow-up scan during ipilimumab plus bevacizumab therapy. While larger baseline tumor diameter was strongly associated with shorter survival, changes of diameter or density, or responses by three criteria did not predict survival. The role of density changes in evaluating response during ipilimumab and bevacizumab therapy for advanced melanoma remains to be further established.

## Background

Recent advances in the understanding of the mechanisms of tumor immunomodulation and the clinical application of immunotherapeutic agents have brought a new era of cancer immunotherapy [1,2]. Clinical benefit of immunotherapeutic agents is best demonstrated in metastatic melanoma, in which ipilimumab, an anti-CTLA-4 antibody, has shown significant improvement in overall survival (OS) [3]. Ipilimumab has shown clinical activity in other solid tumors such as lung cancer and prostate cancer [4-6]. Newer agents, including anti-PD-1 (programmed cell death protein 1) antibodies and anti-PD-L1 (programmed cell death protein ligand-1) antibodies, have also shown marked activity against melanoma and other advanced cancers [7-10], further expanding the role of cancer immunotherapy.

In efforts to further enhance the efficacy of these agents that block immune checkpoint, predictive markers of response to immunotherapy are being actively investigated. The immunosuppressive microenvironment of the tumor may restrict the anti-tumor activity of cancer treatment, which may be further enhanced by the abnormal tumor vasculature [11]. Vascular endothelial growth factor (VEGF) is a potent angiogenic factor that regulates angiogenesis and at the same time increases proliferation, migration, and metastasis of melanoma. VEGF is also known to inhibit dendritic cell maturation and T-cell responses [12,13], thus suppressing antitumor immune responses. Serum level of VEGF-A prior to treatment was shown to be associated with clinical response and OS in advanced melanoma patients treated with ipilimumab which confirmed a generalizable mechanism to immunotherapy resistance via angiogenic cytokines including VEGF [14]. There was no correlation between changes in VEGF levels following treatment and clinical outcome [14]. The finding led to the phase 1 study of the combination therapy of ipilimumab and bevacizumab (anti-angiogenic agent which inhibits VEGF-A). The trial demonstrated a disease-control rate (defined as the proportion of patients with best response of complete response, partial response, or stable disease at any time while on study) of 67.4%. The median survival of this phase 1 study was 25.1 months, which was longer compared to 10.1 months in advanced melanoma patients treated with ipilimumab alone in a prior phase 3 study [3], providing a basis for further pursuit of the combination of immunotherapy and anti-angiogenic therapy [15].

Tumors treated with immunotherapeutic agents are known to demonstrate unique response patterns on imaging, because these agents exert anti-cancer activity by blocking intrinsic immune inhibition by cancer and causing T cell infiltration of the tumors [1-3]. These immune-related response patterns may not be captured by conventional tumor response criteria, such as RECIST and WHO criteria [16,17]. Immune-related response criteria (irRC) have been proposed to better describe treatment results of immunotherapy, and the efforts have been made to further optimize the methods for immune-related response assessment [18,19].

Tumors treated with anti-angiogenic therapy may benefit from incorporation of tumor density change on computed tomography (CT) measured in Hounsfield Unit (HU), as a marker for devascularization and necrosis in response to therapy [20-22]. Furthermore, diameter changes smaller than the conventional threshold may represent response in these patients. Choi criteria defined response as ≥10% diameter decrease or ≥15% decrease in density in patients with gastrointestinal stromal tumors treated with imatinib, which correlate with disease-specific survival [20-22]. In 40 GIST patients treated with imatinib, 32 patients met the Choi response criteria of either a more than 10% decrease in maximum diameter or a more than 15% decrease in tumor density at 2 months after treatment, and these 32 patients had significantly longer time to tumor progression compared to the remaining 8 patients without Choi response [22]. Choi criteria have been used in renal cell carcinomas (RCC) and hepatomas treated with sunitinib that blocks multiple receptor tyrosine kinases including VEGF receptors [23,24]. Among 26 advanced HCC patients, 17 patients (65.4%) were responders by Choi criteria and had a significantly longer TTP (7.5 months) compared with nonresponders (4.8 months; HR = 0.33, P = 0.0182).

In RCC treated with anti-angiogenic therapy, another modified criteria called MASS (morphology, attenuation, size, and structure) criteria has been proposed, and define response as ≥20% diameter decrease, or ≥40HU density decrease, or marked central necrosis in predominantly solid enhancing lesion(s) [16,25]. MASS criteria were recently studied in metastatic melanoma treated with bevacizumab with or without interferon, and shown to strongly predict progression-free survival (PFS) and OS [26]. Given these prior observations and the recent promising phase 1 trial results, it is worthwhile to study tumor diameter and density changes, in addition to conventional tumor diameter changes, during the combined therapy of ipilimumab and bevacizumab in capturing tumor response and predicting outcome.

The purpose of the study is to investigate the tumor diameter and density changes on CT in advanced melanoma patients treated with ipilimumab plus bevacizumab, compare response rates at the first follow-up based on different response criteria incorporating tumor diameters and density, and study association between these measures and survival.

## Results and discussion

A total of 59 measurable lesions in 21 patients (median and mean number of lesions per patient: 2 and 2.8, respectively; range: 1–8) were included. Table 1 summarizes demographics and disease characteristics of the 21 patients. There were 15 lung lesions, 14 peritoneal or retroperitoneal lesions, 11 liver, 9 subcutaneous, 5 nodes and 5 adrenal lesions.

**Table 1:** **Summary of patient characteristics and tumor measurements**

**Baseline demographics and disease characteristics**		
**Gender**	Male	14 (66.7)
	Female	7 (33.3)
**Age (years)**	Median [Range]	53 [25–68]
**Tumor stage**	IV	20 (95.2)
	III Unresectable	1 (4.8)
**Doses of agents**	Ipi: 10 mg/kg, Bev: 15 mg/kg	8 (38.1)
	Ipi: 10 mg/kg, Bev: 7.5 mg/kg	5 (23.8)
	Ipi: 3 mg/kg, Bev: 15 mg/kg	2 (9.5)
	Ipi: 3 mg/kg, Bev: 7.5 mg/kg	6 (28.6)
**Baseline sum diameter (mm)**	Median [Range]	38 [10–178]
	Mean [SD]	55.1 [43.9]
**Baseline average density (HU)**	Median [Range]	43.2 [8.0-69.2]
	Mean [SD]	43.4 [16.2]
**Changes from the baseline measurements**		
**Absolute diameter change at 1** ^**st**^ **follow-up (mm)**	Median [Range]	0 [−21.0 – 56]
	Mean [SD]	5.5 [18.0]
**Proportional diameter change at 1** ^**st**^ **follow-up (%)**	Median [Range]	0 [−64.7 – 85.7]
	Mean [SD]	3.6 [34.4]
**Absolute density change at 1** ^**st**^ **follow-up (HU)**	Median [Range]	−4.3 [−24.3 – 18.5]
	Mean [SD]	−3.1 [10.8]
**Proportional density change at 1** ^**st**^ **follow-up (%)**	Median [Range]	−8.4 [−44.3 – 49.1]
	Mean [SD]	−3.1 [24.5]

The numbers represent the number of patients with percentage in parentheses, unless otherwise specified.

Ipi = ipilimumab, Bev = bevacizumab.

### Lesion-based analysis

**Figure 1:**
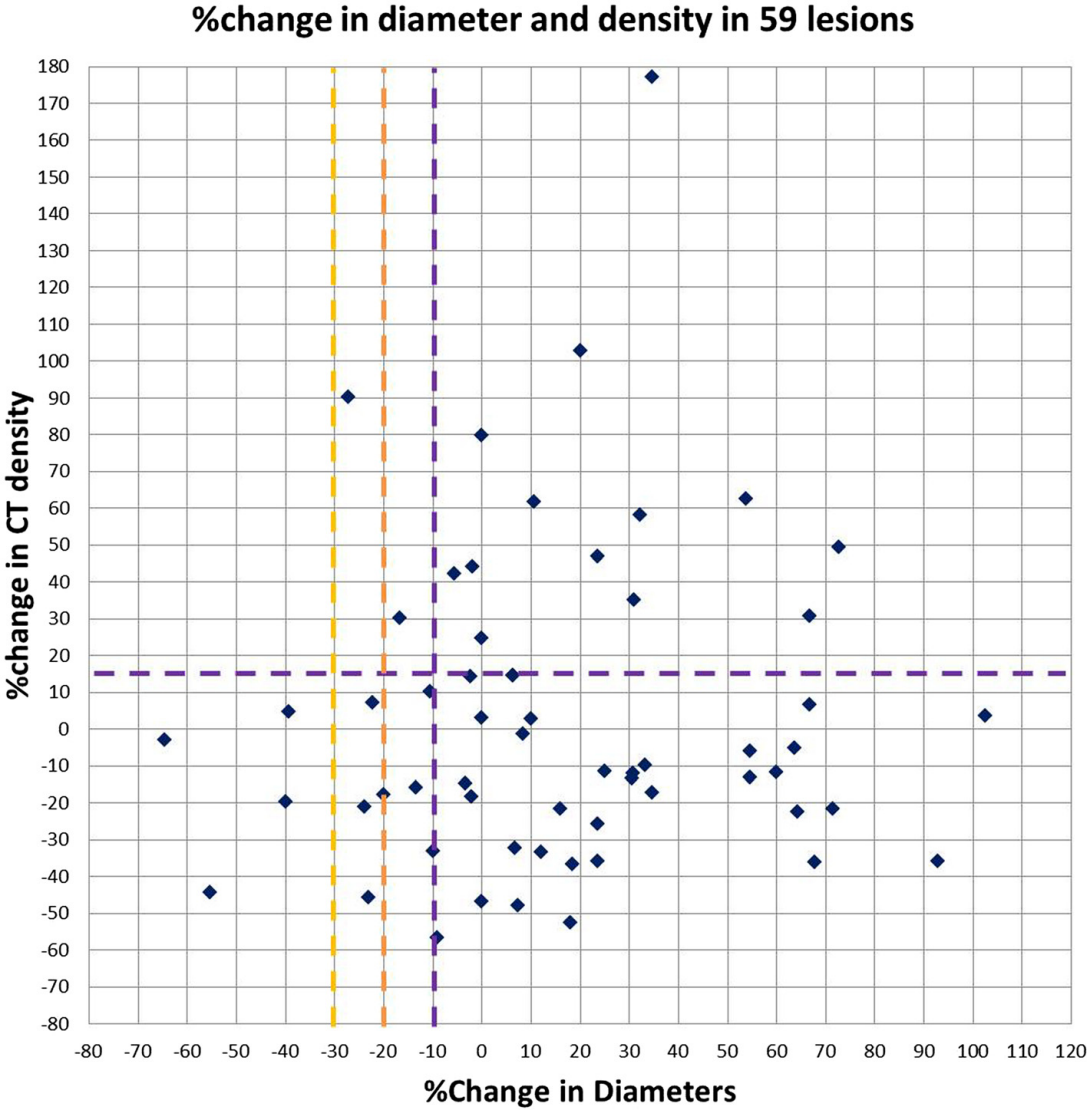
**Scatter plot of the percent changes of the tumor diameter and density on the first follow-up CT compared to the baseline CT in 59 lesions.** The cut-off values for response are indicated by the yellow dashed line for RECIST (≥30% diameter decrease), by the orange dashed line for MASS (≥20% diameter decrease), and by the purple dashed lines for Choi (≥10% diameter decrease or ≥15% density decrease) criteria. No lesions met the density response criteria by MASS.

The median baseline diameter and density for the 59 lesions were 25 mm (range: 10–55) and 44.9 HU (range: 7.2-80.1). The median changes at the first follow-up were 10.7% (range: −64.7 to 102.6) for diameter, and −9.7% (range: -56.6 to 177.2) and −2.7HU (range: -31.0 to 28.3) for CT density. Figure 1 demonstrates the percent changes of diameter and density in 59 lesions. Table 2 summarizes the response by diameter and density of these lesions. No lesions met the density criteria by MASS, while 23 (39%) lesions met the Choi density criteria (≥15% density decrease). When diameter and density changes were combined, 4 lesions (7%) responded by RECIST, 9 lesions (15%) responded by MASS, and 29 lesions (49%) responded by Choi criteria.

**Table 2:** **The number of lesions meeting each response criteria**

**Criteria**	**Number of lesions (%)**
**Diameter changes**	
RECIST (≥30% decrease)	4 (7%)
MASS (≥20% decrease)	9 (15%)
Choi (≥10% decrease)	13 (22%)
**Density changes**	
MASS (≥40HU decrease or marked central necrosis)	0 (0%)
Choi (≥15% decrease)	23 (39%)
**Combined diameter and density criteria**	
MASS (≥20% diameter decrease, ≥40HU density decrease, or marked central necrosis)	9 (15%)
Choi ((≥10% diameter decrease of ≥15% density decrease)	29 (49%)

### Patient-based analysis

**Figure 2:**
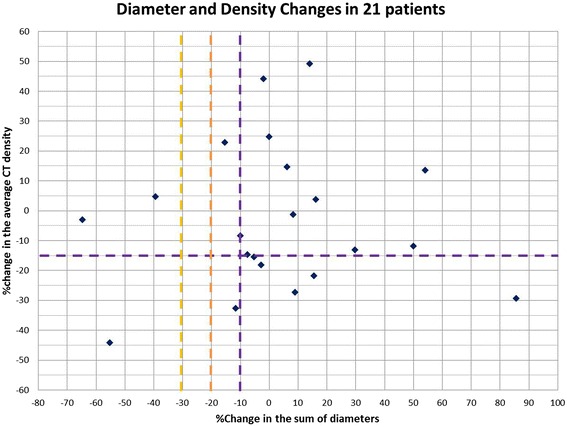
**Scatter plot of the percent changes of the tumor diameter and density on the first follow-up CT compared to the baseline CT in 21 patients.** The cut-off values for response are indicated by the yellow dashed line for RECIST (≥30% diameter decrease), by the orange dashed line for MASS (≥20% diameter decrease), and by the purple dashed lines for Choi (≥10% diameter decrease or ≥15% density decrease) criteria. No patients met the density response criteria by MASS.

**Figure 3:**
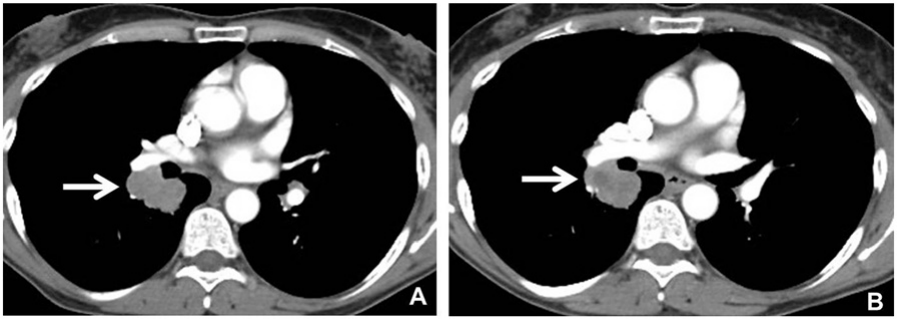
**A 61-year-old male with metastatic melanoma.** Contrast-enhanced CT scan of the chest at baseline **(A)** demonstrate a lobulated right lower lobe mass measuring 37 mm and 54.3 HU (A, arrow). The follow-up scan **(B)** at 11.4 weeks of ipilimumab and bevacizumab therapy demonstrated the lesion measuring 36 mm and 44.4 HU (B, arrow). While the percent decrease of diameter was only 2%, CT density decreased by 18% comparing to baseline, meeting the Choi response criteria.

The baseline sum diameter and average density and their changes on the first follow-up scans are summarized in Table 1. Figure 2 demonstrates the percent changes of diameter and density in 21 patients. No patients met the density criteria by MASS, while 7 (33%, [95% CI: 15-57%]) patients met density response criteria by Choi (Table 3). When diameter and density changes are combined, 3 patients (14%, [95% CI: 3-36%]) responded by RECIST and MASS, and additional 8 patients responded by Choi criteria, resulting in a total of 11 Choi responders (52%, [95% CI: 30-74%]) (Figure 3). None of the patients with response by these criteria developed new lesions at the time of the first follow-up scan. In 2 out of 13 patients with 2 or more lesions, discrepant CT density changes among lesions of the same patient was noted, with some lesions showing >15% density decrease and other lesions showing marked (i.e., >30%) increase in density. Both patients met the response by Choi density criteria when the average density was used to represent the overall change.

**Table 3:** **The number of patients meeting each response criteria**

**Criteria**	**Number of patients (% [95% CI])**
**Diameter changes**	
RECIST (≥30% decrease)	3 (14% [3-36%])
MASS (≥20% decrease)	3 (14% [3-36%])
Choi (≥10% decrease)	6 (28% [11-52%])
**Density changes**	
MASS (≥40HU decrease or marked central necrosis)	0 (0%)
Choi (≥15% decrease)	7 (33% [15-57%])
**Combined diameter and density criteria**	
MASS (≥20% diameter decrease, ≥40HU density decrease, or marked central necrosis)	3 (14% [3-36%])
Choi (≥10% diameter decrease of ≥15% density decrease)	11 (52% [30-74%])

### Association with survival

At the time of analysis, 14 patients (67%) had progressed and 6 patients (29%) had died. The median follow-up was 29.7 months.

#### Baseline diameter/density vs. survival

**Figure 4:**
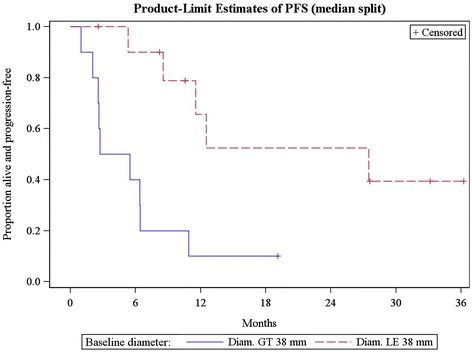
**Progression-free survival in patients dichotomized using the median baseline diameter (38 mm).**

**Figure 5:**
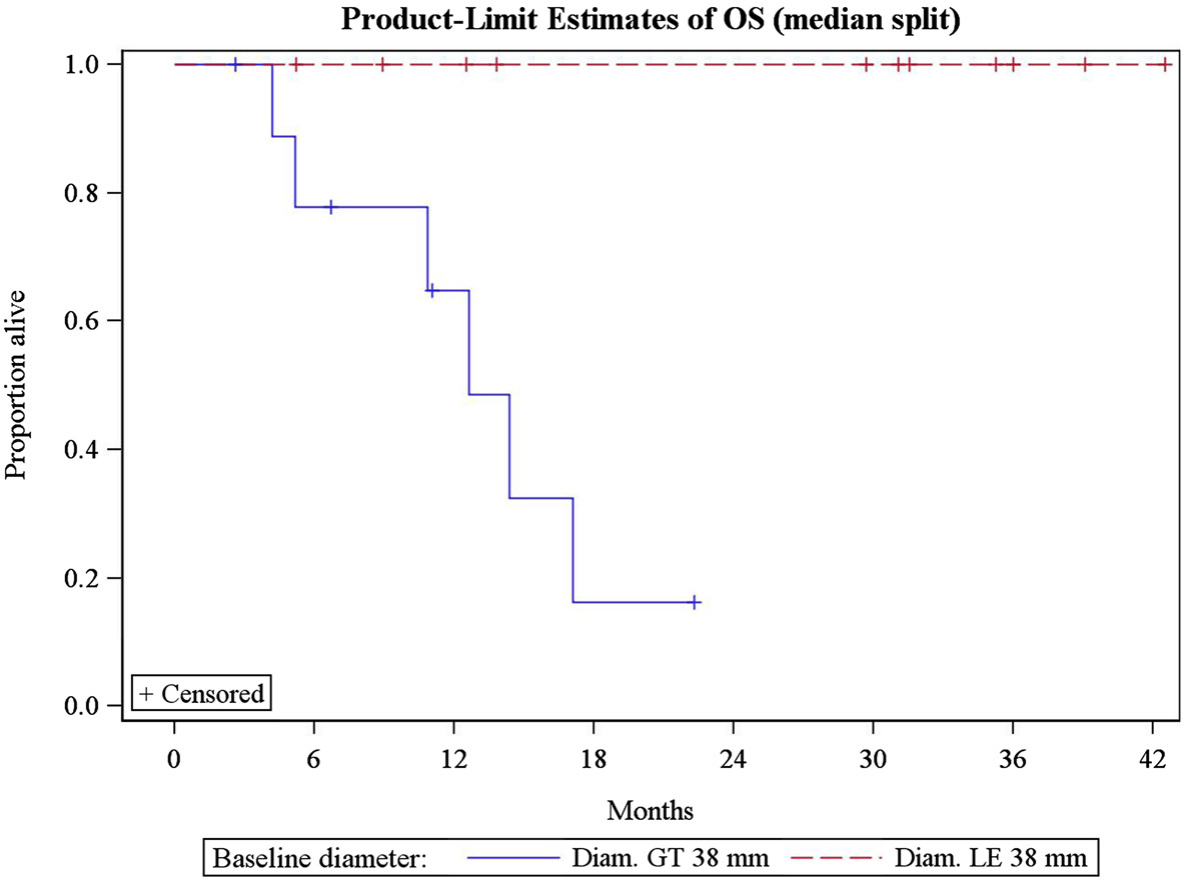
**Overall survival in patients dichotomized using the median baseline diameter (38 mm).**

Baseline diameter was significantly associated with PFS and OS, with larger baseline diameters having poorer outcomes. When patients were dichotomized at the median baseline diameter, which was 38 mm in this cohort, median PFS for patients with baseline diameter ≤38 mm was 27.5 months compared with 4.1 months for those with diameter >38 mm (HR (high vs. low) 5.3, [95% CI 1.6 to 18], p = 0.007). Each 5 mm increase in baseline diameter increased the hazard of a PFS event by 14% (HR: 1.14, [95% CI 1.05-1.23], p = 0.001) (Figure 4). Median OS was not reached for patients with diameter ≤38 mm vs. 12.6 months for those with diameter >38 mm. No deaths occurred in patients with baseline diameters at or below the median. Each 5 mm increase in baseline diameter increase the hazard of death by 18% (HR = 1.18, [95% CI: 1.06-1.31], p = 0.003) (Figure 5).

Baseline density was not associated with PFS (HR (high vs. low) 1.3, [95% CI 0.4 to 3.6], p = 0.68) or with OS (HR (high vs. low) 0.7, 95% CI 0.1 to 4.0, p = 0.71).

#### Diameter/density changes on the 1^st^ follow-up and outcome

Among the total of 21 patients, 5 patients had progressed at the 1^st^ follow-up scan while 16 patients had not progressed. These 16 patients were assessed using a conditional landmark analysis at 11 weeks (the median time to the first scan in this group: 11.3 weeks; range: 11–12 weeks). The continuous Cox model, although limited by the small number of patients (N = 16), suggested that the percent increase of tumor diameter on the 1^st^ follow-up scan result in shorter PFS and OS. Each 5% increase in diameter results in a 13% increase in the hazard of a subsequent PFS event (HR = 1.13, [95% CI: 0.94-1.36], p = 0.18). Each 1% increase in percent change in diameter increased the risk of death 1.5 times (HR = 1.49, [95% CI: 0.8-3.0], p = 0.25). Neither the percent nor absolute change in CT density influenced subsequent PFS (HR for each 5% density increase: 0.997 [95% CI: 0.88-1.14], p = 0.96; HR for each 1 HU density increase: 0.999 [95% CI: 0.94-1.06], p = 0.98) or OS (HR for each 5% density increase:1.08 [95% CI: 0.82-1.44], p = 0.57; HR for each 1 HU density increase: 1.05 [95% CI: 0.91-1.22], p = 0.51).

#### Response by RECIST, MASS and Choi criteria vs survival

RECIST and MASS criteria yielded the same 3 responders and were summarized together. No significant PFS or OS differences were observed between RECIST/MASS responders vs. non-responders (p = 0.45, 0.46, respectively). There was no significant PFS or OS difference between Choi responders vs. non-responders (p = 0.90, 0.94, respectively).

#### Measurement variability

**Figure 6:**
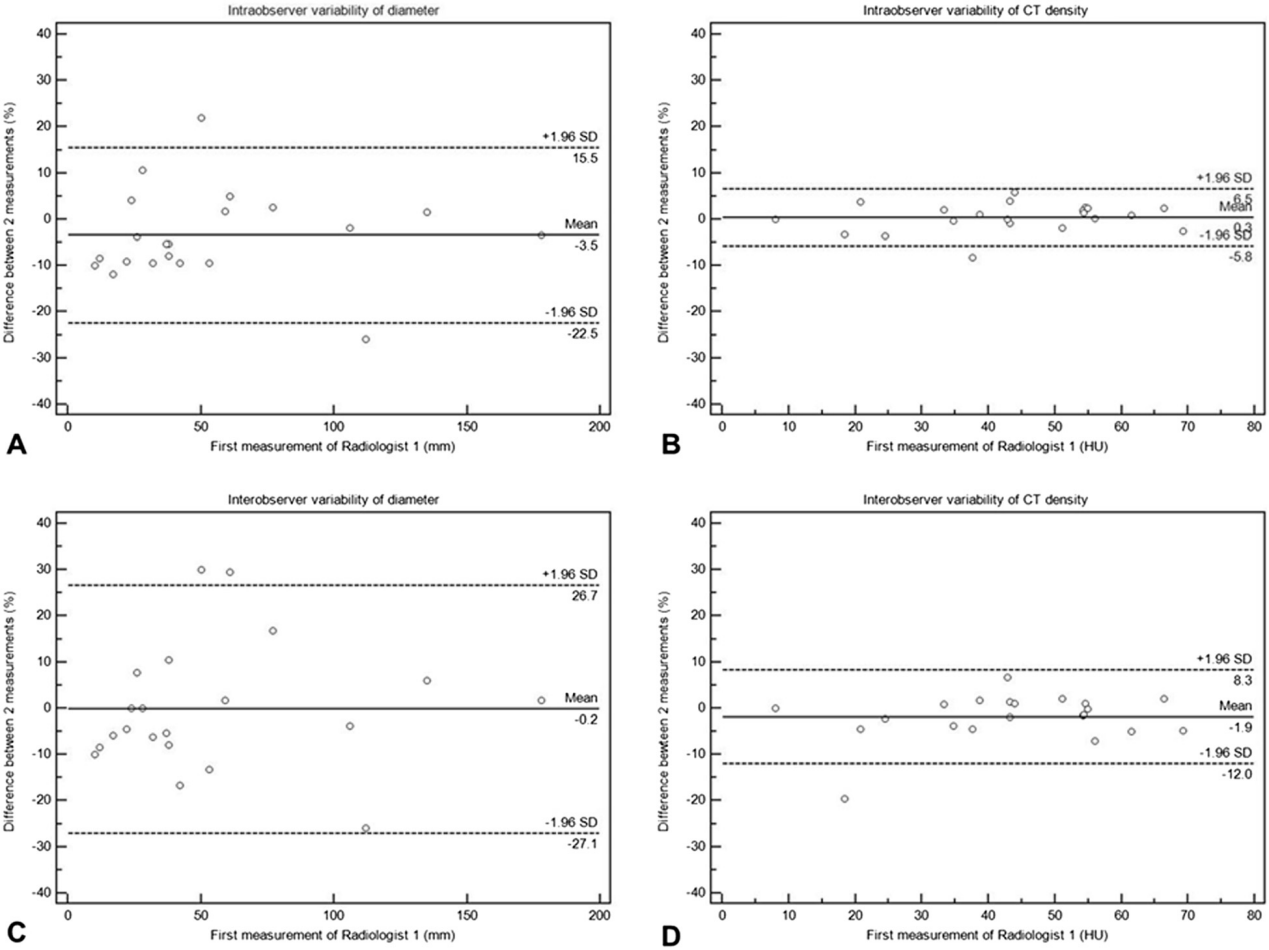
**Intra- and inter-observer variability of diameter and density measurements.** Bland-Altman plots demonstrate the variability of density and diameter measurements (**A**, **B** for intra-observer, **C**, **D** for inter-observer, respectively). The relative difference (%) in two independent measurements for each patient is plotted against the first measurement by Radiologist 1. The straight lines represent the mean relative difference (%), and the dotted lines represent the upper and lower 95% limits of agreement (%).

The intra-observer agreement was high for both diameter and density measurements, with CCC of 0.9865 and 0.9967, respectively. The 95% limits of agreement were (−22.5, 15.5%) for the sum diameter, and were (−5.8, 6.5%) and (−2.3, 2.7HU) for the average density (Table 3, Figure 6A-B). The inter-observer agreement was high for both diameter and density measurements, with CCC of 0.9774 and 0.9934, respectively. The 95% limits of agreement were (−27.1, 26.7%) for the sum diameter, and were (−12.0, 8.3%) and (−4.1, 2.8HU) for the average density (Table 4, Figure 6C-D).

**Table 4:** **Intra- and inter-observer variability of measurements in 21 patients**

**Intra-observer variability**			
	CCC [95% CI]	Mean relative difference	95% limits of agreement
Diameter	0.9865 [0.9691 - 0.9941]	−3.5 (%)	−22.5, 15.5 (%)
CT density	0.9967 [0.9921 - 0.9987]	0.3 (%)	−5.8, 6.5 (%)
		0.2 (HU)	−2.3, 2.7 (HU)
**Inter-observer variability**			
Diameter	0.9774 [0.9459 - 0.9907]	−0.2 (%)	−27.1, 26.7 (%)
CT density	0.9934 [0.9841 - 0.9973]	−1.9 (%)	−12.0, 8.3 (%)
		−0.6 (HU)	−4.1, 2.8 (HU)

## Discussion

The present study demonstrated that ≥15% tumor density decrease by Choi criteria was noted in one third of the advanced melanoma patients treated with ipilimumab plus bevacizumab combination therapy at their first follow-up CT. Larger baseline diameter was strongly associated with shorter PFS and OS, however, diameter and density changes or responses by RECIST, MASS, or Choi criteria at the first follow-up were not associated with survival. While density decrease is a relatively common phenomenon in advanced melanoma treated with ipilimumab plus bevacizumab, further studies are needed to define its role in assessing anti-cancer activity and therapeutic benefit of the agents and to identify objective imaging marker that can predict outcome during the combined immunotherapy and anti-angiogenic therapy.

The degree of diameter and density changes in our cohort were similar to the previous report by Gray et al. in their study of metastatic melanoma patients treated with bevacizumab with or without interferon, which reported the average of 2% diameter change and −7% density change [26]. No lesions or patients in our study met the density response criteria by MASS, indicating that such a marked decrease in density is a rare phenomenon among melanoma patients receiving ipilimumab plus bevacizumab. Our observation is similar with the report by Gary et al., which had only 1 out of 118 lesions showing marked central necrosis [26]. Density decrease ≥40HU were more frequent in their cohort (6/118 lesions (5%) and 6/44 patients (14%)), which could be due to the different therapeutic regimen in the prior report where the majority (39/44, 89%) of the patients received interferon in addition to bevacizumab [26].

When three different criteria for response were used for 59 lesions, the lesion-based response rate was 7% (4/59) by RECIST, 15% (9/59) by MASS, and 49% (29/59) by Choi criteria. For the patient-based analysis, the response rate was 14% (3/21) by RECIST and MASS, and 52% (11/21) by Choi (4 patients responding by diameter decrease, 5 patients by density decrease and 2 patients meeting both diameter and density criteria). The increase in response rate by applying the Choi density criteria indicates that CT density decrease may be a sequela of the anti-cancer activity of ipilimumab and bevacizumab therapy. Similar increase of response rate was noted in the prior study, in which response rates at the first follow-up CT were 7% (3/44) for RECIST, 14% (6/44) for MASS, and 34% (15/44) for Choi criteria [26].

Heterogeneous changes of CT density within same patient were noted in 15% (2/13) among the patients with more than 1 lesion, while overall assessment using the average density met Choi density response criteria in both patients. Tumoral heterogeneity is an important issue in assessing response to targeted therapy [27,28], and the quantitative imaging approach to address this issue remain to be established. The current standard approach including the one used in the present study relies on a certain number of representative lesions to demonstrate systemic tumor burden changes, which is associated with inherent limitations. Further studies are needed to assess the frequency and impact of heterogeneous density changes during therapy.

While different definitions of response can give rise to different rates of response, these modified definitions of response need to be validated by studying association with outcome. The alternate definitions are of great clinical significance if they can differentiate responders with survival benefit more accurately than the conventional definitions. In our study, baseline diameter was the only significant predictor of PFS and OS; other measures including baseline density and diameter/density changes at the first follow-up were not significantly associated with survival. Univariate Cox models suggested that the percent increase of tumor diameter on the 1^st^ follow-up scan may result in shorter PFS and OS; however, these results need to be viewed cautiously given the small number of patients and events. None of the three response criteria (RECIST, MASS and Choi criteria) differentiated patients with longer survival at the first follow-up scan, indicating the need to further studies to identify objective markers that can predict survival at the early course of therapy to guide therapeutic decisions. Given the unique mechanism of anti-cancer activity of ipilimumab, the density changes in the present cohort may be at least in part due to infiltration of tumor by immune cells. Future investigations may also focus on the biological background of the density changes, as well as the comparison of tumor density among cohorts receiving ipilimumab alone, bevacizumab alone and the combination.

Tumor density changes have been extensively studied in the context of anti-angiogenic therapy to improve strategy for tumor response evaluation [20,21,23,25]. Recently, immune-related responses have been investigated based on tumor size changes [17-19,29]. The present study represents the first attempt to further optimize the existing tumor response criteria specifically for combined therapy using anti-angiogenic agents and immunomodulating agents, which will be more frequently used in treatment of advanced cancer in the near future.

Gary et al. reported that MASS response at the first follow-up strongly predicted PFS and OS [26]. The different results between 2 studies may be due to the different regimen. Our study also had only 3 MASS responders. High baseline serum lactate dehydrogenase (LDH) level was also associated with survival in their study [26]. In our cohort, stratification according to elevated baseline LDH was not possible since there were only 2 patients with elevated levels. The association between baseline measures and survival was not mentioned in the prior study [26].

Our study demonstrated high intra- and inter-observer agreement for both diameter and density measurements. Based on the 95% limits of agreement, 15% density decrease was beyond the intra- and inter-observer measurement variability in our cohort. However, 10% diameter decrease was within the 95% limits of intra- and inter-observer agreement, alerting the possibility of misclassification by measurement error when applying Choi criteria [30]. Intra-observer variability was narrower than inter-observer variability for both diameter and density, indicating the measurements by same reader on baseline and follow-up scans help to decrease misclassification. Given nearly two thirds of Choi responders (7/11, 64%) responded by density criteria regardless of the diameter changes, it may worthwhile to see if adding the density criteria to the conventional RECIST diameter criteria (≥30% decrease) may better identify patients with therapeutic benefit while avoiding misclassification.

The limitations of the present study include retrospective design and a small number of patients treated at a single institution. Due to the design of the phase 1 trial, the doses of ipilimumab and bevacizumab varied among the patients in the small cohort. The study reports the initial observations of tumor diameter and density changes during ipilimumab and bevacizumab therapy, which needs to be studied further in larger cohorts. The study also focused on the tumor changes at the first follow-up study; the role of serial measurements of diameter and density in defining progression and treatment failure remain to be investigated. The serial CT density measurements may also help to identify cases with delayed response to immunotherapy. In addition, the serial measurements will provide an opportunity to assess the impact of immune-related response assessment incorporating new lesions into the measurements in comparison with the conventional RECIST based approach in the assessment of CT tumor density.

## Conclusions

In conclusion, tumor density decrease meeting Choi criteria (≥15 % decrease) was relatively common during ipilimumab plus bevacizumab combination therapy for advanced melanoma, noted in one-third of the patients. Larger baseline tumor diameter was strongly associated with shorter survival; however, diameter and density changes at the first follow-up or responses by RECIST, MASS or Choi criteria were not associated with survival in these patients. The role of density changes in evaluating anti-cancer activity and therapeutic benefit of these agents remain to be further studied in a larger cohort.

## Methods

### Patients

The study included 21 advanced melanoma patients (14 males, 7 females; median age: 53 years, age range: 25–68) treated in a phase 1 trial of ipilimumab plus bevacizumab at the Dana-Farber Cancer Institute [15]. All patients had baseline CT and at least one follow-up CT using iodinated intravenous contrast agents, and had at least one measurable lesion (≥10 mm longest diameters for non-nodal lesions, ≥15 mm in short axis for lymph nodes [31,32]). Patients were treated with ipilimumab with four doses at 3-week intervals and then every 12 weeks, and bevacizumab every 3 weeks [15]. The protocol was approved by the Institutional Review Board of the Dana-Farber Cancer Institute, and all patients provided written informed consent. The clinical trial results including survival and adverse events of the entire multicenter cohort have been previously reported [15].

### CT tumor measurements

The standard clinical protocol for body CT at the Dana-Farber Cancer Institute used a 64-row MDCT scanner (Aquilion 64; Toshiba America Medical Systems, CA). Patients are scanned in the supine position from the cranial to caudal direction from the clavicles to the pubic symphysis at end-inspiration. During the study, 100 mL of iopromid (Ultravist 300, 300 mg iodine/mL; Bayer HealthCare Pharmaceuticals Inc. Wayne, NJ) is injected intravenously at a rate of 3 mL/sec, with a scan delay of 30 seconds for chest and 70 seconds for abdomen (portal venous phase). Axial images (5 mm thickness) were reconstructed and transferred to a Picture Archiving Communication System (PACS) workstation (Centricity, General Electric, Milwaukee,WI).

Baseline CT scans prior to initiation of therapy (median time between baseline scan and initiation of therapy: 1.0 week; range: 0.3-3.0 weeks) were retrospectively reviewed by a board-certified radiologist with expertise in oncologic imaging (M.N.). All measurable lesions in each patient were selected, regardless of the number of lesions in total or per organ, in order to evaluate heterogeneity among lesions within the same patient [28]. The exception included 2 patients with innumerable (>20) lesions in one organ (lung in one patient and liver in other), in whom the largest 5 lesions within the organ (lung/liver) were selected, in addition to all the measurable lesions in other organs.

The diameters (mm; the longest diameters for non-nodal lesions and short axis for nodes) and density (HU) were measured for all lesions on contrast-enhanced CT images on baseline scans and on the first follow-up scans (median time to the first follow-up scan: 11.3 weeks). Diameters were measured using a caliper-type measurement tool on PACS workstation [33]. The CT attenuation was measured using an oval region of interest covering the maximum area of each lesion excluding the surrounding structures [34]. Three lesions (2 lung and 1 subcutaneous lesions) demonstrated <0 HU at baseline due to partial volume effects, which were ineligible for the study and excluded.

### Diameter and density changes on follow-up

The percent changes of diameter and CT density were calculated on the follow-up scan in reference to the baseline [35,36]. For CT density, the absolute change (HU) was also calculated. For lesion-based analysis, the diameter and density measurements of each lesion were used. For patient-based analysis, the sum of the diameters and the average of CT density were used to represent baseline and follow-up measurements [20-22,36]; for those who had >5 lesions in total and >2 lesions per organ, up to 5 largest lesions in total and up to 2 largest lesions per organ were chosen according to RECIST1.1, based on the baseline measurements.

Response was assigned for each lesion and each patient, based on RECIST (≥30% decrease in diameter) [31,32,37], MASS (≥20% decrease in diameter or ≥40HU decrease in density or marked central necrosis) [25,38] and Choi criteria (≥10% decrease in diameter or ≥15% decrease in density) [20-22] (Table 5).

**Table 5:** **Summary of tumor response assessment and definitions for RECIST, Choi and MASS criteria**

	**RECIST**	**MASS**	**Choi**
**Measurements and lesion evaluations**	Diameter^*1^	Diameter	Diameter
		CT attenuation (HU)	CT attenuation (HU)
		Marked central necrosis^*2^	
**Response criteria** ^*3^	≥30% diameter decrease	≥20% diameter decrease, ≥40HU decrease, or marked central necrosis	≥10% diameter decrease, or ≥15% density decrease

*1: The longest diameters for non-nodal lesions and short axis for nodes were used according to RECIST1.1 [31]. This was applied to size measurement for Choi and MASS criteria in the present study.

*2: Marked central necrosis is defined as >50% of the enhancing central portion of a predominantly solid enhancing mass subjectively changing to near fluid attenuation (necrosis) after treatment [25].

*3: The sum of the diameters of all target lesions and the average of CT density measured in HU are used to assess response, comparing the values on the follow-up scan during therapy in reference to the values on the baseline pretherapy scan.

### Measurement variability

To assess measurement variability, two board-certified radiologists (Radiologist 1: M.N. and Radiologist 2: N.H.R.) independently measured the diameter and density of all lesions on baseline scans, without access to other radiologist’s measurements, as described previously [18,19,36,39]. Radiologist 1 performed measurements twice with one week interval, without access to the prior measurements.

### Statistical analysis

Descriptive methods were used to summarize patient demographic and disease characteristics. Measurements on a continuous scale were summarized using mean, median, standard deviation, and range. Categorical characteristics were summarized using percentages and 95% exact binomial confidence intervals. The distributions of progression-free survival (PFS) and overall survival (OS) were assessed using the product-limit method of Kaplan–Meier, with 95% confidence intervals (CI) estimated using log [−log (outcome)] methodology. To investigate the association between baseline diameter/density and outcome, PFS and OS were compared between 2 groups dichotomized at the median baseline diameter (38 mm) or density (43.2 HU). Cox proportional hazards models were used to estimate hazard ratios (HRs) and 95% confidence intervals; p-values are based on the Wald chi-squared statistic. In addition, univariate Cox proportional hazard models were used to estimate the effects on outcome of 5 mm increments in baseline diameter or 5 HU increments in baseline density. Eleven-week conditional landmark analyses were used to evaluate differences in outcome according to response or changes in diameter or density.

All p-values were two-sided, with statistical significance defined as P <0.05. There were no corrections for multiple comparisons.

Intra- and inter-observer variability were assessed using concordance correlation coefficients (CCCs), mean relative difference and 95% limits of agreement. CCCs are products of a measure of precision and a measure of accuracy where CCC value 1 indicates perfect agreement and −1 indicates perfect reversed agreement [40]. The mean relative difference (%) between the two measurements is defined as 100*[M_1_-M_2_]/M_1_ (M_1_ = measurement 1, M_2_ = measurement 2). Bland-Altman plots were used to visually demonstrate the variability between the two measurements [36,39,41]. Two measurements of Radiologist 1 were used to assess intra-observer variability. The first measurement of Radiologist 1 and the measurement by Radiologist 2 were used to evaluate inter-observer variability.

Both survival and measurement variability were assessed according to patient-based analyses, using the sum diameters and the average density for each patient.

## Abbreviations

OS: Overall survival
PD-1: Program death-1
VEGF: Vascular endothelial growth factor
RECIST: Response evaluation criteria in solid tumors
WHO: World Health Organization
irRC: Immune-related response criteria
CT: Computed tomography
HU: Hounsfield unit
RCC: Renal cell carcinomas
MASS: Morphology, attenuation, size, and structure
PFS: Progression-free survival
LDH: Lactate dehydrogenase
MDCT: Multi-row detector CT
PACS: Picture Archiving Communication System
CI: Confidence intervals
HR: Hazard ratios
CCC: Concordance correlation coefficient
GT: Greater than
LE: Less than or equal to
